# Avalanche Transients of Thick 0.35 µm CMOS Single-Photon Avalanche Diodes

**DOI:** 10.3390/mi11090869

**Published:** 2020-09-19

**Authors:** Bernhard Goll, Bernhard Steindl, Horst Zimmermann

**Affiliations:** Institute of Electrodynamics, Microwave and Circuit Engineering, TU Wien, Gusshausstrasse 25/E354-02, A-1040 Wien, Austria; bernhard.steindl@tuwien.ac.at (B.S.); horst.zimmermann@tuwien.ac.at (H.Z.)

**Keywords:** single-photon avalanche diode (SPAD), gating, avalanche transients, 3.3 V/0.35 µm complementary metal-oxide-semiconductor (CMOS)

## Abstract

Two types of single-photon avalanche diodes (SPADs) with different diameters are investigated regarding their avalanche behavior. SPAD type A was designed in standard 0.35-µm complementary metal-oxide-semiconductor (CMOS) including a 12-µm thick p^-^ epi-layer with diameters of 50, 100, 200, and 400 µm; and type B was implemented in the high-voltage (HV) line of this process with diameters of 48.2 and 98.2 µm. Each SPAD is wire-bonded to a 0.35-µm CMOS clocked gating chip, which controls charge up to a maximum 6.6-V excess bias, active, and quench phase as well as readout during one clock period. Measurements of the cathode voltage after photon hits at SPAD type A resulted in fall times (80 to 20%) of 10.2 ns for the 50-µm diameter SPAD for an excess bias of 4.2 V and 3.45 ns for the 200-µm diameter device for an excess bias of 4.26 V. For type B, fall times of 8 ns for 48.2-µm diameter and 5.4-V excess bias as well as 2 ns for 98.2-µm diameter and 5.9-V excess bias were determined. In measuring the whole capacitance at the cathode of the SPAD with gating chip connected, the avalanche currents through the detector were calculated. This resulted in peak avalanche currents of, e.g., 1.19 mA for the 100-µm SPAD type A and 1.64 mA for the 98.2-µm SPAD type B for an excess bias of 5 and 4.9 V, respectively.

## 1. Introduction

Avalanche photodiodes (APDs) operated with a reverse voltage larger than the breakdown voltage (Geiger mode) are usually capable of detecting single photons and are termed single-photon-avalanche-diodes (SPADs). A photon absorption generates an electron-hole pair, and in combination with the high-electric field in a multiplication zone, a large avalanche with charge carriers might be triggered due to impact ionization. This causes a current, which discharges the SPAD until its cathode-anode voltage reaches the breakdown voltage, where the avalanche is quenched. For a further detection of a photon, the SPAD has to be recharged again.

With reference to [1], the avalanche build-up (time between electron-hole pair generation due to a photon hit and reaching maximum avalanche charge) action of a SPAD consists at first in a local charge multiplication, a local voltage drop to breakdown level and then a spreading to lateral directions. After build-up, final quenching happens. The spreading might take place with the help of charge carriers, which move towards side directions or create additional secondary photons when some avalanche carriers recombine. The simplest equivalent circuit to model an avalanche action is the capacitance of the reverse biased avalanche diode in parallel with a resistor [1,2]. When a photon enters a SPAD, the photon detection probability (PDP) describes the chance that a self-sustaining avalanche is triggered. Even in the absence of photons, dark counts occur in a SPAD, which are uncorrelated avalanches due to thermal-/trap-assisted carrier generation or tunneling. They are characterized by a mean dark count rate (DCR). Afterpulses, on the other hand, are avalanches, which are correlated to a previous avalanche. There are many reasons for afterpulses, e.g., the release of a carrier by a deep-level trap, which has been filled during a previous current flow, or, e.g., the diffusion of secondary charge carriers into the high-field zone of the SPAD, which were generated by photons originating from recombination during avalanche current flow. The probability for appearance of an afterpulse is described with afterpulsing probability (APP). It becomes lower the more time elapses after a previous avalanche. The voltage difference between how much the diode’s reverse-voltage is higher than the breakdown voltage is one of the main parameters in operating a SPAD and is called excess bias. Typically, DCR, APP, and PDP increase when raising the excess bias [3,4,5,6]. After an avalanche has happened in a SPAD, it needs a dead time, which is controlled by surrounding circuitry, until it is recharged again and ready for a new photon detection. The APP strongly depends on the dead time. If the dead time is longer, the APP will decrease.

SPADs are important in applications like photon detectors for quantum communications [7,8] and quantum random number generators [9,10,11]. Recently, many multi-pixel image sensors with SPADs were published [12,13,14,15,16,17], some for 3D imaging. SPAD arrays have potential for highly sensitive optical data receivers [18,19,20,21]. Typically, the bit error rate (BER) of SPAD receivers suffers from DCR and APP. Forward error correction might be a solution to solve this problem [22]. A BER of 2 × 10^−3^ is sufficient to use a concatenated Reed–Solomon code super-forward-error-correction (FEC) scheme to get a BER better than 10^−9^ with 6.69% redundancy (ITU-T G.975.1). In [23], a 64 × 64 SPAD array in 130 nm complementary metal-oxide-semiconductor (CMOS) was capable to receive a 500 Mb/s 4-PAM optical signal with −46.1 dBm sensitivity for a bit error rate (BER) of 2 × 10^−3^ when using equalization. In [24], five subsequent time slots (one period of a 250 MHz clock), where each slot consists of the information of whether one or no photon was detected from a gating circuit in a 3.3-V/0.35-µm CMOS technology with one SPAD are fed into a shift register. Hence it could be decided whether a bit has been received or not with the knowledge of how many detections happened in a row of five time slots. This fully integrated optical receiver achieved a data rate of 50 Mb/s in non-return-to-zero (NRZ) with a sensitivity of −57 dBm (BER = 2 × 10^−3^).

This paper presents measurement results of the cathode voltage drop of two types of SPADs with different diameters when an avalanche occurs. SPAD type A was fabricated in standard 0.35-µm CMOS with 12-µm thick p^-^ epi-layer with active diameters of 50, 100, 200, and 400 µm and type B was implemented in the high-voltage (HV) line of this process with active diameters of 48.2 and 98.2 µm. With the knowledge of the measured capacitance at the cathode node, the avalanche currents through each SPAD were determined. The voltage transient response with a measurement of its 80 to 20% fall time of SPAD type A with 50 µm diameter was already published in [25]. Each SPAD is wire-bonded to a clocked gating chip, which controls charge up to maximum 6.6-V excess bias, active, and quench phase as well as readout during one clock period. This cascaded gating chip was designed in a standard 3.3-V/0.35-µm CMOS technology.

## 2. Single-Photon Avalanche Diodes (SPADs)

**Figure 1:**
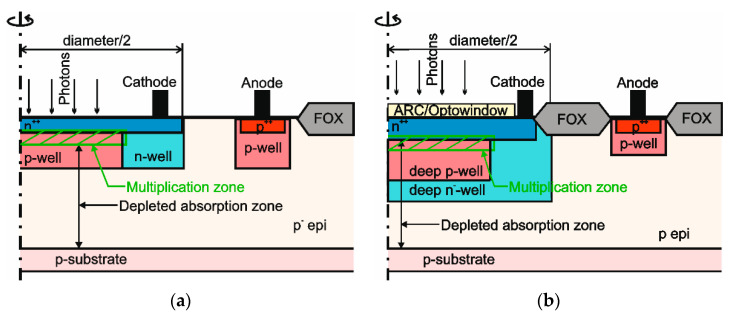
Cross sections (not to scale) of (**a**) single-photon avalanche diode (SPAD) type A in 0.35 µm standard CMOS with p^-^ epi-layer and (**b**) SPAD type B in 0.35 µm high-voltage (HV) complementary metal-oxide-semiconductor (CMOS) with standard p epi-layer, deep p-well, and deep n^-^-well for high-voltage applications (FOX = field oxide).

In Figure 1, cross sections of SPAD type A (see Figure 1a) and SPAD type B (see Figure 1b) are depicted. Both diodes were fabricated in a 3.3-V/0.35-µm CMOS technology. Type A had a ≈12-µm thick low-doped p^-^ epi layer with a doping concentration of ≈2 × 10^13^/cm^3^ and type B had the epitaxial layer of the high-voltage process version, which was doped with ≈10^15^/cm^3^ [26]. Each type of SPAD could be integrated together with additional circuitry on one chip each [24] when the electronic part was isolated from the substrate with the help of deep n-wells. SPAD type A used the standard CMOS process line and consisted of a n^++^ cathode with a p-well below to form a high-field multiplication zone. A thick p^-^ epi layer acted as an absorption zone with a high-enough electric field for a high drift velocity of photo-generated charge carriers. Therefore, the maximum sensitivity of the SPAD was located in the visible red and was very near infrared region of the spectrum of the light. An n-well around the multiplication zone prevented the SPAD from edge breakdown. SPAD type A was fabricated in diameters of 50, 100, 200, and 400 µm. Typical values for its DCR were, e.g., ≈10^4^ counts per second (CpS) and for APP, e.g., 0.2% for a diameter of 50 µm at 20 °C, an excess bias of 3 V and a dead time of 9.5 ns [5]. The value of the PDP amounted to 21.5% for a wavelength of 635 nm [24], where the excess bias typically was near below ≈3 V for best BER. For a diameter of 100 µm, the DCR amounted to 1.89 × 10^4^ CpS and 3.08 × 10^4^ CpS for an excess bias of 3.3 and 6.6 V, respectively, at a temperature of 25 °C [27]. The APP was 0.7 and 4.8% for an excess bias of 3.3 and 6.6 V, respectively, and a dead time of 9.5 ns. For an excess bias of 6.6 V and wavelengths of 635 and 850 nm, PDPs of 35.1 and 22%, respectively, were achieved.

SPAD type B was designed in the high-voltage (HV) line of this 0.35-µm CMOS technology with the option of an oxide opening (opto window) with anti-reflection coating (ARC) above the diode (see Figure 1b), which was a nitride layer, where the thickness was optimized for no reflection at visible red light. The cathode consisted of a highly doped n^++^ region, which was slightly thicker than the cathode of type A. The high-field multiplication zone was located at the passage between cathode and a deep p-well. To assure that the electric field in the depleted absorption zone, which has the same function as for SPAD type A, was high enough for a high drift velocity of photo-generated charge carriers, a deep n^-^-well was added. SPAD type B was fabricated in diameters of 49.2 and 98.2 µm. At a temperature of 25 °C and for a diameter of 49.2 µm, the best measured DCR amounts to 2.88 × 10^4^ CpS for samples in the middle of the wafer up to 14.04 × 10^4^ CpS for samples from the border for an excess bias of 6.6 V. The APP was determined to be near 80% for samples in the middle and down to 10% for samples from the border of the wafer if a dead time of 5.8 ns was used [28]. The PDP was 37.4, 27.9, and 18.6% for wavelengths of the light of 780, 850, and 900 nm, respectively. For the SPAD type B with a diameter of 98.2 µm, a DCR of ≈ 5.5 × 10^5^ CpS for an excess bias of 3.3 V at a temperature of 25 °C was measured [29]. The APP was ≈ 10% for an excess bias of 3.3 V and 6 ns dead time. The measured PDP for an excess bias of 3.2 V was ≈21% for 650 nm wavelength. For a wavelength of 800 nm and 6.6 V excess bias, a PDP of 35% was obtained.

A figure of merit (FoM) to compare the performance of different detectors is the noise-equivalent power (NEP) [28,30]. It is depicted in Equation (1), where *h* is the Planck constant, *c* is the speed of light in vacuum, and *λ* the wavelength of the used light.

(1) $$NEP\;=\;\frac{hc}{\lambda }\frac{\sqrt{2DCR}}{PDP}$$

For SPAD type A with a diameter of 50 µm, this resulted to *NEP* ≈ 205.8 aW√Hz for 3-V excess bias and 635-nm wavelength. For SPAD type B with a diameter of 49.2 µm, a best *NEP* ≈ 163.4 aW√Hz was calculated for 6.6-V excess bias and 780-nm wavelength. The focus of this paper is the transient measurement of photon-triggered avalanche pulses. More information about PDP, DCR, and APP (also in dependence on excess bias) of SPADs type A and B is published in [5,28,29].

## 3. Gating Chip

For controlling the SPADs, a gating chip was designed in 0.35-µm CMOS technology with a nominal supply voltage of 3.3 V. In comparison to a quenching circuit, a gated SPAD has defined, mostly periodic time slots, where it is set to active and ready for photon detection. Once a photon has triggered an avalanche, the SPAD is conducting until the breakdown voltage is reached by the cathode-anode voltage or until the reset phase starts (if the photon was absorbed close to the end of the active phase) and quenches the detector below breakdown voltage. This can be, e.g., done with a clock signal, which defines that in one half of the clock period the SPAD is active and in the other half it is quenched. In the active time window, at most, one avalanche can occur. Therefore, to be able to detect more photons, the clock frequency has to be increased. In the case of a data receiver, this results in a larger clock frequency than the data rate [24].

**Figure 2:**
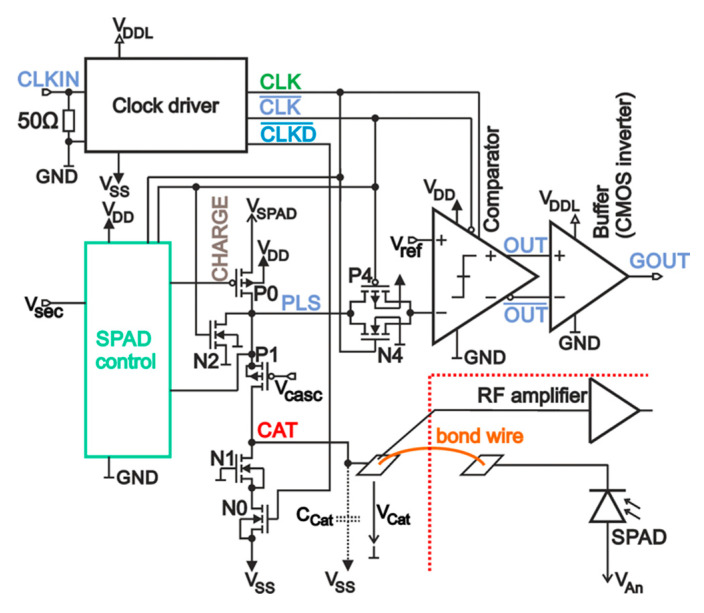
Block diagram of a clocked gating control chip, where a SPAD is connected with a bond wire. A radio frequency (RF) amplifier measures the cathode voltage via a RF probe needle on the pad at node cathode node (CAT).

**Figure 3:**
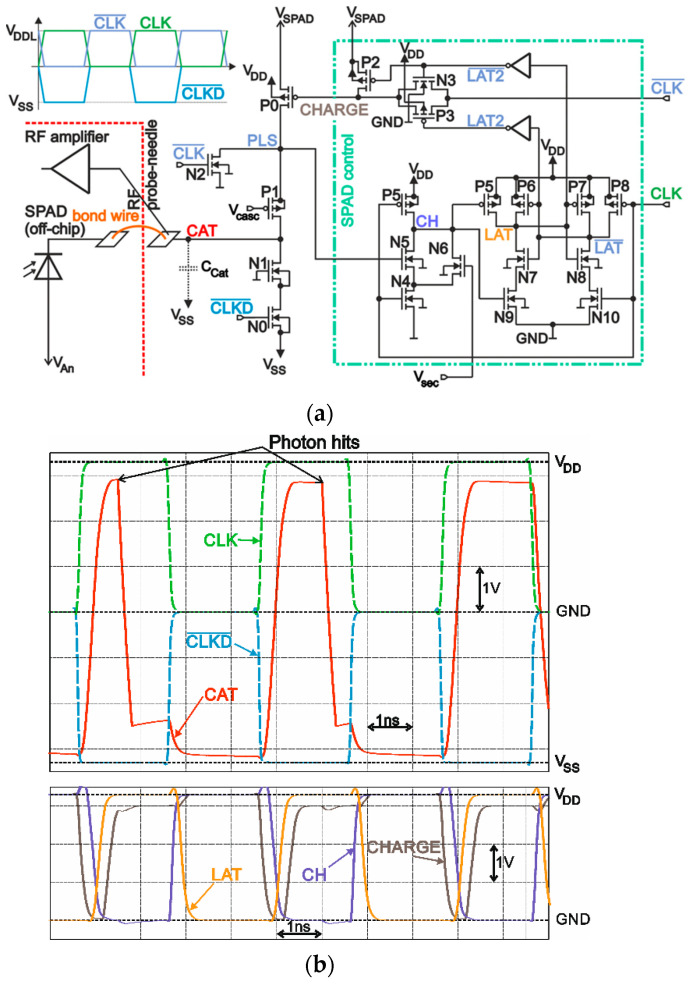
SPAD control in the gating control chip: (**a**) Schematics; (**b**) Simulated transients assuming a fast responding SPAD. A photon hit triggers an avalanche in the SPAD, which discharges node CAT until the cathode-anode voltage reaches its breakdown voltage and the avalanche is quenched.

Figure 2 shows the block diagram of the gating control chip. The cathode of the SPAD was bonded with gold wire with 25-µm diameter and 1-mm length to the node CAT. Hence the gating controller can pull the cathode potential to ≈*V_SPAD_* = 3.3 V to set the SPAD active for photon detection in Geiger mode or to ≈*V_SS_* = −3.3 V to quench the SPAD in the reset phase. The resulting cathode-anode voltage is *V_SPAD_*-*V_An_* for detection and *V_SS_*-*V_An_* for reset. For operation, the breakdown level of the SPAD should be located somewhere in between. A detailed schematic of the whole gating control chip including the transients are depicted in Figure 3. On-chip, a clock driver generated digital clocks for the circuit block SPAD control and for the switching transistors out of a sine wave with ≈600 mV amplitude, which was applied to pad CLKIN. With a bond wire, CLKIN was connected to a 50-Ω micro-strip line on the printed circuit board (PCB). Therefore, an on-chip 50-Ω resistor was added for termination without appreciable reflections. The on-chip clock driver generates a digital non-inverted and inverted clock, nodes CLK, and $\bar{\mathrm{CLK}}$, where the logical voltage levels were ground node GND = 0 V for digital low and *V_DDL_* = 3.3 V for digital high. Clock signal $\bar{\mathrm{CLKD}}$ corresponded to $\bar{\mathrm{CLK}}$, but was level shifted down by 3.3 V so that the logical voltage levels result to −3.3 and 0 V.

The anode voltage of the external SPAD was applied via a separated pad, which was wire-bonded to the PCB. To charge up the cathode node (CAT) to ≈*V_SPAD_* = 3.3 V, transistor P0 was turned on. To discharge node CAT to ≈*V_SS_* = −3.3 V for quenching an avalanche in the SPAD, or to deactivate it, transistor N0 was switched on. All transistors are specified to work with a supply voltage of 3.3 V. To be able to switch node CAT between ≈*V_SS_* = −3.3 V and ≈*V_SPAD_* = 3.3 V, which resulted into a 6.6-V swing, cascode transistors P1 and N1 were added to protect all transistors against overvoltage. N-MOS transistors are typically smaller and faster with less parasitic capacitances than P-MOS transistors. Therefore, it was sufficient to connect the gate of N1 to GND, whereas on the gate of P1, a voltage of *V_cas_*_c_ = −1 V was applied to faster charge-up node CAT and to reduce voltage peaks in the drain-source voltage of P0 and P1, which could exceed their critical voltages during switching action. On the other side, transistor N2 helped discharging node PLS due to similar reasons.

A clock period consisted of a reset phase (CLK = $\bar{\mathrm{CLKD}}$ = GND = 0 V and $\bar{\mathrm{CLK}}$ = *V_DDL_* = 3.3 V), where the cathode-anode voltage of the SPAD was below the breakdown voltage, when node CAT was pulled down to ≈*V_SS_* = −3.3 V, *PLS* ≈ 0 V, and an active phase where CLK switches to *V_DDL_* = 3.3 V ($\bar{\mathrm{CLK}}$ = GND = 0 V and $\bar{\mathrm{CLKD}}$ = *V_SS_* = −3.3 V). In reset of the SPAD, the node voltages in the unit SPAD control were $\bar{\mathrm{LAT}}$ = CH = *V_DD_* = 3.3 V and *LAT* = 0 V, thus transmission gate P3/N3 was turned on, P2 was off, and node CHARGE = $\bar{\mathrm{CLK}}$ = *V_DDL_* = 3.3 V, hence transistor P0 was off. Transistor N0 was turned on.

In the active phase, transistor N0 is off. At first, a small fraction of the time duration in this phase was used to charge up the SPAD in pulling up node CAT and PLS to ≈*V_SPAD_* = 3.3 V with P0 turned on, because at the beginning, nodes $\bar{\mathrm{LAT}}$ = CH = V_DD_ and *LAT* = 0 V, thus transmission gate P3/N3 was on, P2 off, and CHARGE = $\bar{\mathrm{CLK}}$ = 0 V. Transistor P0 was charging node CAT and PLS until PLS reached a voltage level near *V_SPAD_*. This charge-up was monitored at node PLS with transistor N5, which is turned on after its gate-source voltage raises above its threshold voltage. As a consequence, node CH is discharged with transistor N4 to ≈ 0 V, which forces the latch to flip to *LAT* = *V_DD_* and $\bar{\mathrm{LAT}}$ = 0 V, thus transmission gate P3/N3 is turned off and P2 is turned on, which charges up node CHARGE to *V_SPAD_* to stop charging up node CAT and PLS with transistor P0. Because of parasitic capacitances and delay times of logic elements, the time lag between detection with N5 and turning off P0 was sufficiently long that nodes CAT and PLS can easily reach a voltage level very close to V_SPAD_. Following this, the cathode-anode voltage was above the breakdown voltage; transistors P0, N0, and N2 were turned off; node CAT was charged up and floating; and the SPAD was ready for photon detection. The excess bias depended on the size of the anode voltage *V_AN_* of the SPAD. For a distinct breakdown voltage *V_BD_*, the excess bias *V_EB_* can be calculated to *V_EB_* ≈ *V_SPAD_*−*V_AN_*−*V_BD_*, where the breakdown level of the cathode voltage must be located between *V_SPAD_* and *V_SS_*, hence *V_EB_* ≤ *V_SPAD_−V_SS_*.

There could be the case that during charge up of node CAT, when P0 is turned on, an avalanche might occur in the SPAD, which would result especially for low ohmic diodes in a large current flow. Consequently, transistor N5 would never detect a finished charge up at node PLS and P0 stays turned on until the subsequent reset phase. To avoid such a large current flow during nearly the whole active phase of the SPAD, transistor N6 was added. With voltage *V_sec_* at the gate of N5, node CH can be discharged independently from transistor N5 during an adjustable time duration. Thus, with adjusting *V_sec_*, transistor N6 can be turned off or a discharging time for node CH can be set.

To read out whether an avalanche occurred or not, transmission gate N4/P4 was turned on during the active phase of the SPAD. The clocked comparator was in reset. In the subsequent reset phase of the SPAD, transmission gate N4/P4 was turned off and the voltage at node PLS was stored dynamically in the parasitic capacitance at the negative input-node of the comparator at the end of the active phase. A voltage drop indicated an avalanche. In the reset phase of the SPAD the comparator compared the stored voltage with a reference voltage *V_ref_* to generate a digital decision at node OUT dependent on whether an avalanche occurred or not. With *V_ref_*, the detection threshold for the voltage drop at PLS can be set. Finally, a 50-Ω driver was implemented, which consisted of a chain of inverters capable to drive an off-chip 50 Ω load, i.e., a fast oscilloscope.

Capacitance *C_Ca_*_t_ in Figure 2 and Figure 3a represents the overall node capacitance of node CAT including the cathode of the SPAD. Neglecting the bond wire in between due to its low inductance was justified. When an avalanche occurs during the active phase, transistors N0, P0 and N2 were off and only the current through the SPAD can discharge node CAT. Consequently, the avalanche current can be calculated out of the transient of the avalanche with *C_Ca_*_t_ × *d*V_Cat_/dt when measuring the whole node capacitance *C_Cat_*, which included the capacitance of the SPAD due to its connection with a bond wire.

## 4. Measurement Setup, Results, and Discussion

Each SPAD was glued together with one gating chip on a printed circuit board (PCB) consisting of FR4 base material (FR = flame retardant). The cathode of the SPAD was bonded with a gold bond wire to node CAT of the gating chip. The anode of the SPAD was bonded to a DC line on the PCB, which provides the negative anode voltage V_AN_. The sine wave to generate on-chip a digital clock signal was applied via a microstrip line and an SMA connector on the PCB to the gating chip. All supply and reference voltages were provided to the chip via connectors, block capacitances, and lines on the PCB as well. The PCB itself was mounted on a copper block with temperature sensor and with a Peltier cooler below, which regulated the temperature on the PCB to 25 °C for measurements.

The transients of the cathode voltage at the pad of node CAT (see Figure 1) were measured with a high-speed radio frequency (RF) probe, Picoprobe Model 35 from GGB Industries, which had a frequency response from DC to 26 GHz, an operating range of −6 to 6 V, 10:1 signal attenuation, and 1.25-MΩ and 50-fF load at the input. The Model 35 Picoprobe was connected with a coaxial cable (K-system) to a Keysight MSOV204A Mixed Signal Oscilloscope with 20 GHz bandwidth and at maximum 80-GSa/s sampling rate. Unfortunately, the anode pad of the SPAD was situated nearby the cathode pad. Therefore the needle was placed on the pad of node CAT to avoid an accidental touch to the anode pad with its large negative voltage, and consequently, to damage of the expensive Picoprobe. The length of the bond wire between the cathode of the SPAD and node CAT of the gating chip was ≈2 mm, which corresponds to a series inductivity of ≈2 nH when using 1 nH/mm as a rule of thumb. A comparison of the transient at node CAT with the transient directly at the cathode pad of the SPAD revealed no noteworthy difference.

**Figure 4:**
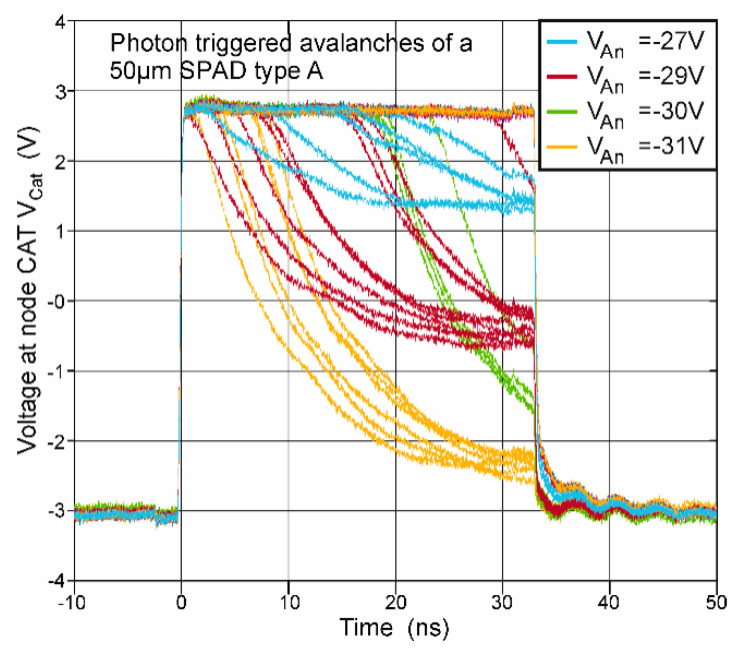
Transients of the cathode voltage (node CAT) of a SPAD type A with a diameter of 50 µm for different anode voltages *V_AN_*. In the illustration, transients of several active phases are overlaid. It can be seen that early avalanches quench (themselves) during the active phase, when the breakdown level is reached. Some avalanches, which occurred later in the active phase are quenched by the following reset phase. The observed breakdown level in this figure depends on the anode voltage *V_AN_*. It can be calculated with the breakdown voltage as *V_AN_* + *V_BD_*.

For measuring the transient signals, the gating chip was clocked with 15 MHz, which results in a duty cycle of 50% for a time duration of the active phase of the SPAD of ≈33.3 ns to be able to observe the whole discharging phase when an avalanche occurs. The minimum dead time corresponded to the time duration of the reset phase of the SPAD, which amounted to ≈33.3 ns. Figure 4 shows typical results of the cathode voltage’s transient with and without avalanche events, where several active phases have been overlaid for illustration.

If an avalanche occurs, e.g., due to a photon hit, node CAT is discharged by the avalanche current of the SPAD. Avalanches, which happen earlier in the active phase have enough time to discharge node CAT until the breakdown level is reached. Later avalanches are quenched by the following reset phase. If no avalanche occurs, the voltage at node CAT remains and will be only switched below breakdown level during the reset phase.

**Figure 5:**
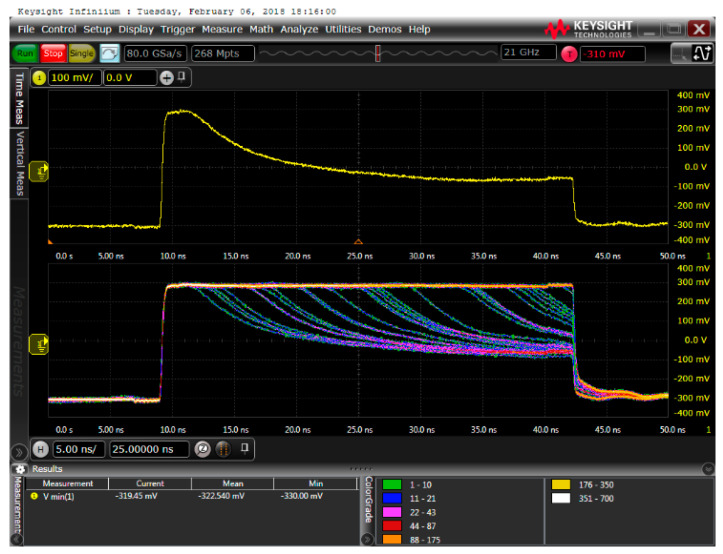
Oscilloscope picture of one avalanche (**top**) and several overlaid avalanches (**bottom**) of the cathode voltage of a SPAD type B with 48.2 µm diameter for an illumination power with a halogen source to achieve somewhat equally distributed photon hits.

With lots of samples of avalanche transients, which happened approximately in the first quarter of the active phase, an 80 to 20% fall time could be measured for the different SPADs. For this, the SPAD was illuminated with a halogen light so that the photon hits on the SPAD were somewhat equally distributed over the time duration of the active phase when overlaying several cycles with a storage oscilloscope as, e.g., depicted in Figure 5 for a SPAD type B with 48.2-µm diameter for an anode voltage of −66 V. When considering an ideal exponential decay of the excess bias, the time constant τ can be calculated by dividing the 80 to 20% fall time by ln(4).

**Figure 6:**
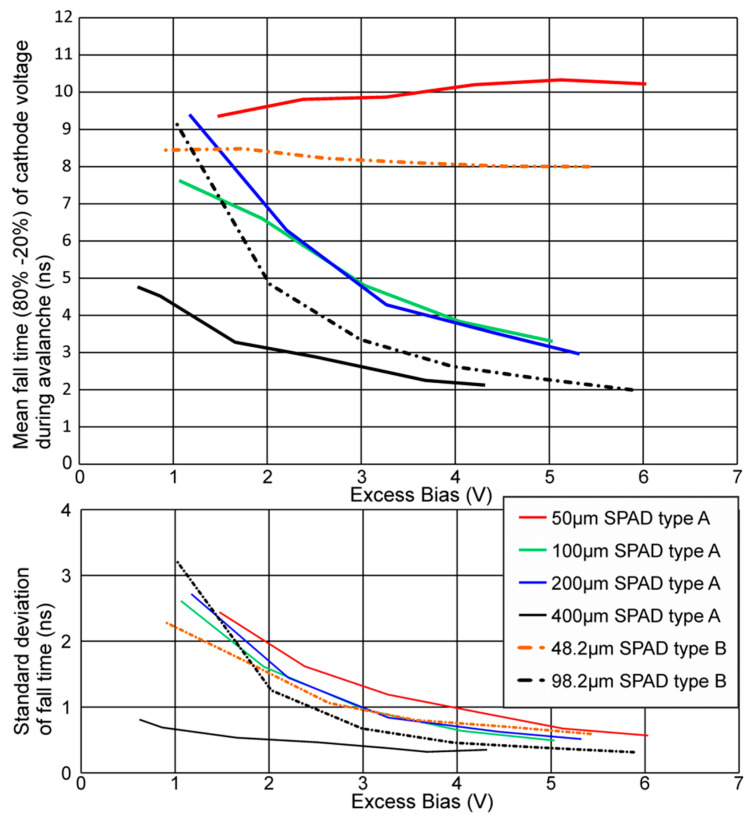
80 to 20% fall time of the cathode voltage of different SPADs when an avalanche occurred.

Figure 6 shows the results for the different types of SPADs. There is a tendency that SPADs of type B in the HV CMOS technology are somewhat faster than SPADs of type A due to more avalanche current. The reason could be that for SPAD type B in HV CMOS technology deeper wells with a tendency to lower doping are used to meet requirements of high-voltage operation, which increases the breakdown voltage. This may result in a vertical thinner p epi layer (thinner absorption zone) below the SPAD type B, which in combination with the deep n-well increases somewhat the field in the depleted absorption zone. For SPADs with a diameter larger than 98.2 µm, a drop of the fall time was observed (meaning the avalanche build-up is faster) when the excess bias was increased, where the avalanche current got larger.

This was because the size of the SPADs’ capacitance dominates over the pad capacitances. For the SPAD type A with 50 µm and SPAD type B with 48-µm diameter, the fall time remained approximately constant, because the pad and parasitic capacitances dominated. Concluding, the measurement shows a tendency to a lower fall time for larger SPADs.

**Figure 7:**
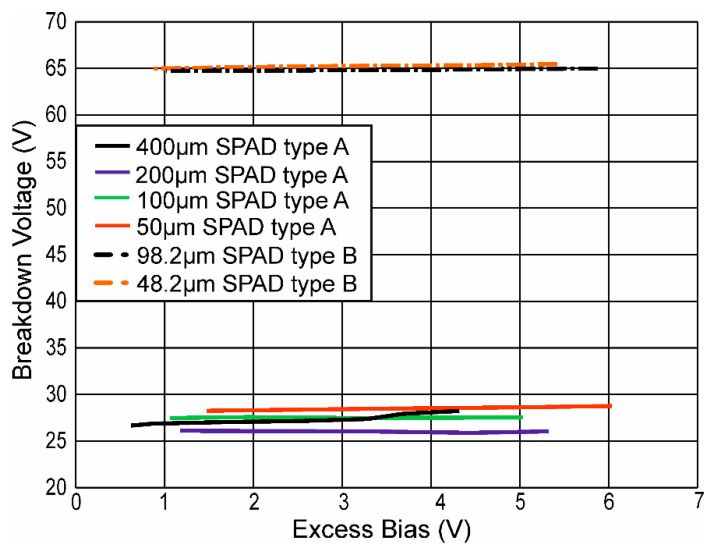
Breakdown voltage vs. excess bias.

For those avalanche transients, which could reach the breakdown level before the end of the active phase, the breakdown voltage *V_BD_* was determined from the breakdown level *V_BL_* from the relationship *V_BD_* = *V_BL_*-*V_AN_*. In Figure 7, the results of the breakdown voltage for several SPADs versus the excess bias are depicted. All breakdown voltages were mostly independent from the excess bias (as expected). The small variation for SPAD type A with 400-µm diameter were originated from the gating chip, which came due to the larger capacitance of the SPAD to its operation limit. The measured breakdown voltages amounted to 28.5, 27.5, 26, and ≈27 V for SPAD type A with 50, 100, 200, and 400 µm, respectively. For SPAD type B breakdown voltages of 65.2 and 64.8 V, they were determined for diameters of 48.2 and 98.2 µm, respectively.

The avalanche current transient can be calculated, if the overall node capacitance *C_Cat_* is measured and the cathode voltage is evaluated by *C_Cat_* × *d*V_Cat_/dt. The capacitances *C_Cat_* for each SPAD, which was connected with a gating chip, were measured with an Agilent 4284A precision LCR meter. For SPAD type A, the anode voltage was chosen to be as close as possible below breakdown. Due to device restrictions, for SPAD type B, the anode voltage was set to −40 V for capacitance measurements. The results for the SPADs type A including pads and node CAT on the gating chip were 0.84, 1.12, 1.2, and 2.2 pF for the SPAD diameters of 50, 100, 200, and 400 µm, respectively. For SPAD type B, the capacitances 0.88 and 1.12 pF were measured for the diameters of 48.2 and 98.2 pF.

**Figure 8:**
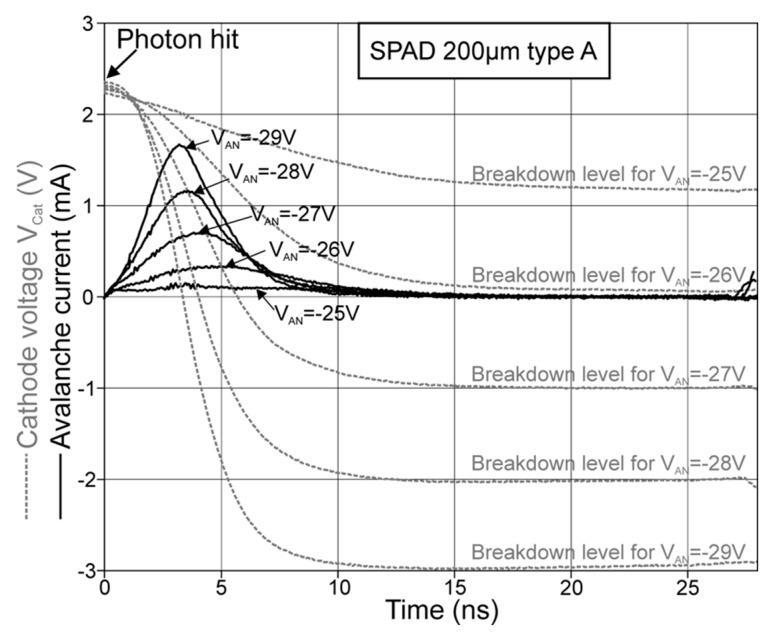
Avalanche-current transients for a SPAD type A with 200 µm diameter for different anode voltages V_AN_, where the avalanche starts at 0 ns.

**Figure 9:**
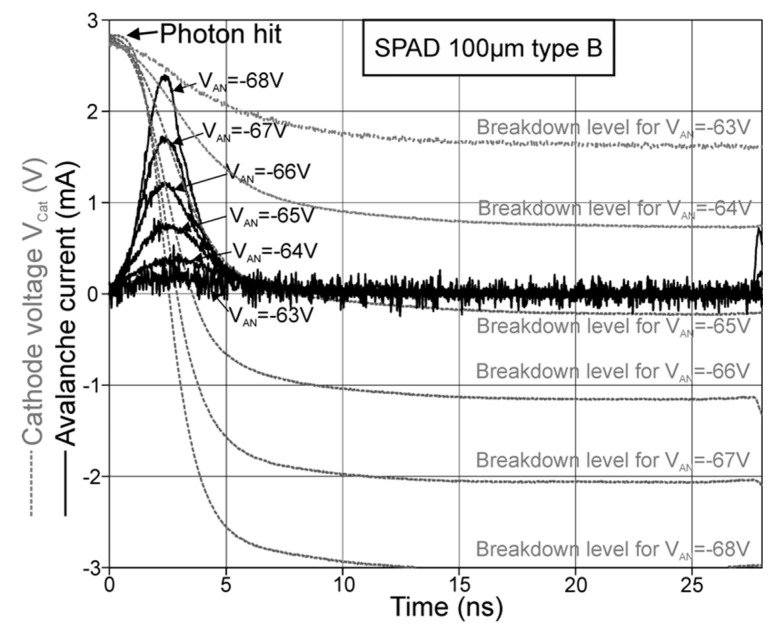
Avalanche-current transients for a SPAD type B with 98.2 µm diameter for different anode voltages V_AN_, where the avalanche starts at 0 ns.

**Figure 10:**
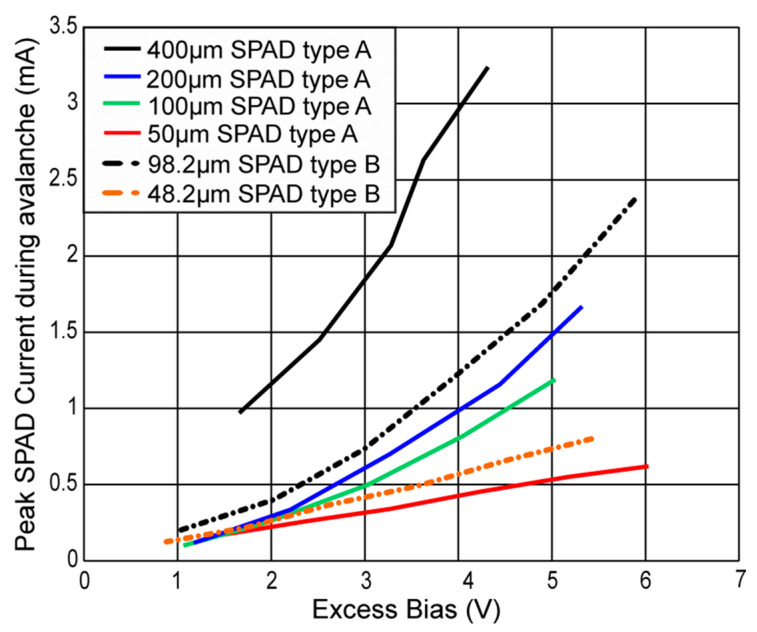
Peak avalanche-current for SPADs type A and B in dependence on the excess bias.

Figure 8 shows the resulting avalanche-current transients for SPAD type A with 200-µm diameter, and in Figure 9, the current transients for SPAD type B with 98.2 µm diameter are plotted. In Figure 8 and Figure 9, the anode voltages V_AN_ of the SPADs are varied, which directly alters the excess bias. It can be seen, that at the beginning of the avalanche, the current raised towards a maximum current value, while the cathode of the SPAD was discharged and the excess bias dropped. This decrease operated against a further growing of the avalanche current, because the ionization coefficients for electrons and holes decline for lower field strengths. For every excess bias, which would be held constant, there exists a static avalanche current after breakdown. This avalanche current is smaller for a lower excess bias and vanishes at the breakdown level of the SPAD, where the excess bias amounts to zero. After the point of a peak avalanche-current, the current decreased, because the cathode’s voltage dropped towards the breakdown level. The peak avalanche currents for all SPADs are plotted in Figure 10 in dependence on the excess bias.

In Figure 8, there is a considerable shift of the peak current towards larger time points from 3 ns to almost 5 ns when changing the anode voltage from −29 to −26 V. For −25 V, noise influences the current transient. In Figure 9 the shift of the peak current occurs to a less extent. This can be explained, if the range of the excess bias from 0 to 6.6 V is seen in relation to the breakdown voltage, which was lower for the 200-µm SPAD type A with 26 V than for the 98.2-µm SPAD type B with 64.8 V. Therefore, the same excess bias caused a larger increase of the electric field in the multiplication region of SPAD type A than in SPAD type B.

For SPADs type A and SPADs type B, the peak avalanche current increased with raising excess bias and larger diameter. This can be observed in the same way in Figure 6, because a larger avalanche current caused a faster discharge and hence a lower fall time. For SPAD type A with a diameter of 50 µm and SPAD type B with a diameter of 48.2 µm, the pad capacitances were dominant and therefore the delay times and avalanche currents were nearly the same in the observed range of the excess bias.

## 5. Conclusions

Knowing the behavior of SPADs helps to develop more detailed models. Especially for fully integrated data receivers, it is important to optimize the circuit to a distinct SPAD.

In this paper, the avalanche actions of two types of single-photon avalanche diodes (SPADs) in 0.35-µm CMOS technology were investigated. SPAD type A with diameters of 50, 100, 200, and 400 µm was designed in standard CMOS technology including a 12-µm thick p^-^ epi-layer. SPAD type B with diameters of 48.2 and 98.2 µm was designed in the high-voltage (HV) line of this technology. Each SPAD was wire-bonded to a clocked gating chip in standard 0.35-µm CMOS technology with a nominal supply voltage of 3.3 V. This chip controls in one clock period the charge up to maximum 6.6 V excess bias in the active phase as well as the quenching and read out in the reset phase. Measurements of the cathode voltage transients after photon hit at SPAD type A resulted in fall times (80 to 20%) of 10.2 ns for 50-µm SPAD diameter and an excess bias of 4.2 V and 3.45 ns for 200-µm SPAD diameter and an excess bias of 4.26 V. For type B fall times of 8 ns for 48.2-µm SPAD diameter and 5.4 V excess bias as well as 2 ns for 98.2-µm SPAD diameter and 5.9 V excess bias were determined. To calculate the avalanche current transients of the SPADs out of the transients of the cathode voltage in using the relationship *C_Cat_* × *d*V_Cat_/dt, the whole capacitance at the cathode of the SPAD including gating chip connected were measured. This may be an alternative method to determine the current during discharging by the SPAD from a high-ohmic cathode node. It resulted in peak avalanche currents in the transients of, e.g., 1.19 mA for 100-µm SPAD type A and 1.64 mA for 98.2-µm SPAD type B for an excess bias of 5 and 4.9 V, respectively.

The breakdown voltages amounted to 28.5, 27.5, 26, and ≈27 V for SPAD type A with 50, 100, 200, and 400 µm, respectively, and 65.2 and 64.8 V for SPAD type B with diameters of 48.2 and 98.2 µm, respectively. Typically, the avalanche current rises for larger excess bias and larger diameter of the SPAD. Consequently, this has an effect on the fall time of the cathode voltage drop, when an avalanche occurs. The fall time depends on the avalanche current and typically gets smaller when the excess bias or the diameter of the SPAD is increased.
